# Ophthalmic complications of endoscopic sinus surgery^☆^

**DOI:** 10.1016/j.bjorl.2016.04.006

**Published:** 2016-05-04

**Authors:** Malgorzata Seredyka-Burduk, Pawel Krzysztof Burduk, Malgorzata Wierzchowska, Bartlomiej Kaluzny, Grazyna Malukiewicz

**Affiliations:** a Nicolaus Copernicus University in Toruń, Faculty of Medicine, Department of Optometry Collegium Medicum, Toruń, Poland; b Nicolaus Copernicus University in Toruń, Faculty of Medicine, Department of Ophthalmology Collegium Medicum, Toruń, Poland; c Nicolaus Copernicus University in Toruń, Faculty of Medicine, Department of Otolaryngology and Laryngological Oncology Collegium Medicum, Toruń, Poland

**Keywords:** Endoscopic sinus surgery, Orbital/ocular, Chronic rhinosinusitis, Cirurgia endoscópica do seio nasal, Orbital/ocular, Rinossinusite crônica

## Abstract

**Introduction:**

The proximity of the paranasal sinuses to the orbit and its contents allows the occurence of injuries in both primary or revision surgery. The majority of orbital complications are minor. The major complications are seen in 0.01–2.25% and some of them can be serious, leading to permanent dysfunction.

**Objective:**

The aim of this study was to determine the risk and type of ophthalmic complications among patients operated due to a chronic rhinosinusitis.

**Methods:**

This is a retrospective study of 1658 patients who underwent endoscopic sinus surgery for chronic rhinosinusitis with or without polyps or mucocele. Surgeries were performed under general anesthesia in all cases and consisted of polyps’ removal, followed by middle metal antrostomy, partial or complete ethmoidectomy, frontal recess surgery and sphenoid surgery if necessary. The ophthalmic complications were classified according to type, frequency and clinical findings.

**Results:**

In our material 32.68% of the patients required revision surgery and only 10.1% had been previously operated in our Department. Overall complications occurred in 11 patients (0.66%). Minor complications were observed in 5 patients (0.3%) with the most frequent being periorbital ecchymosis with or without emphysema. Major complications were observed in one patient (0.06%) and were related to a lacrimal duct injury. Severe complications occurred in 5 cases (0.3%), with 2 cases and referred to a retroorbital hematoma, optic nerve injury (2 cases) and one case of extraocular muscle injury.

**Conclusions:**

Orbital complications of endoscopic nasal surgery are rare. The incidence of serious complications, causing permanent disabilities is less than 0.3%. The most important parameters responsible for complications are extension of the disease, previous endoscopic surgery and coexisting anticoagulant treatment.

## Resumo

**Introdução:**

A proximidade dos seios paranasais à órbita e seu conteúdo tornam possível a ocorrência de lesões tanto na cirurgia primária como na de revisão. A maioria das complicações orbitais são menores. As maiores são observadas em 0,01%-2,25% e algumas delas podem ser graves levando a disfunção permanente.

**Objetivo:**

O objetivo deste estudo foi identificar o risco e o tipo de complicações oftalmológicas em pacientes operados devido a rinossinusite crônica.

**Método:**

Foi realizado um estudo retrospectivo de 1.658 pacientes submetidos a cirurgia endoscópica sinusal devido a rinossinusite crônica com ou sem pólipos ou mucocele. As cirurgias foram realizadas sob anestesia geral em todos os casos e consistiram de remoção de pólipos, seguida de antrostomia meatal média ou etmoidectomia parcial ou completa, cirurgia de recesso frontal e cirurgia de esfenoide se necessário. As complicações oftalmológicas foram classificadas de acordo com o tipo, frequência e achados clínicos.

**Resultados:**

Em nosso material 32,68% dos pacientes necessitaram de cirurgia de revisão e apenas 10,1% haviam sido anteriormente operados em nosso departamento. As complicações gerais ocorreram em 11 pacientes (0,66%). Complicações menores foram observadas em 5 pacientes (0,3%), sendo que a mais frequente foi equimose periorbital com ou sem enfisema. Complicações maiores foram observadas em um paciente (0,06%) e atribuída à lesão do ducto lacrimal. Complicações graves ocorreram em 5 casos (0,3%) e foram referidas como hematoma retrorbital (2 casos), lesão do nervo óptico (2 casos) e um caso de lesão muscular extraocular.

**Conclusões:**

As complicações orbitais da cirurgia endoscópica nasal são raras. A incidência de complicações graves que causam incapacidade permanente é de menos de 0,3%. Os parâmetros mais importantes responsáveis por complicações são extensão da doença, cirurgia endoscópica anterior e tratamento anticoagulante coexistente.

## Introduction

Functional Endoscopic Sinus Surgery (FESS) is the most appropriate surgical procedure for sinus pathology treatment. Over the last decade the procedure developed to relatively safe.^1^, ^2^, ^3^, ^4^ The overall incidence of minor and major complication after FESS is range from 0.4% to 30%.^1^, ^2^, ^5^, ^6^ The anatomy proximity of the paranasal sinuses to the orbits exposes it is to the risk of trauma.^2^, ^6^ The majority of orbital complications are minor ones (3.9–20.24%). The major complications are seen in 0.01–2.25%, but some of them could be serious, leading to permanent dysfunction.^1^, ^2^, ^5^, ^6^, ^7^, ^8^

The ophthalmic complications could be classified as: minor (grade I) included injury to the lamina papyracea, major (grade II) injury to the lacrimal duct and finally serious (grade III) as retroorbital hemorrhage, injury to the optic nerve or any reduction of vision or blindness and injury of orbital muscle.^1^, ^6^, ^9^, ^10^ As the minor and major ophthalmic complications are normally without any permanent disabilities the serious ones are potentially harmful.^1^, ^2^, ^5^, ^6^

To reduce or eliminate the incidence of ophthalmic complications the precise preoperative Computer Tomography (CT), Magnetic Resonance Imagine (MRI), utilization of the Lund–MacKay Index and novel technique are recommended. The even more important thing is training and learning curves experience in FESS surgery.^1^, ^2^, ^11^, ^12^

We analyzed 1658 patients who underwent endoscopic surgery due to inflammatory disease of the paranasal sinuses at our Department over 9 years from 2005 to 2013. The ophthalmic complications were matched with type, frequency and clinical findings.

## Methods

We performed a retrospective study of 1658 patients who underwent FESS for Chronic Rhinosinusitis (CRS) from 2005 through 2013 at the Department of Otolaryngology. The study protocol was reviewed and approved by the Ethic Committee (decision number 366/2015). All patients with CRS with or without polyps and patients with mucoceles were included in the study. The patients with diagnosed benign and malignant tumors were excluded from the study. The diagnosis of CRS was made in accordance with history and objective findings. The cases were graded according to the Lund MacKay score and the surgical extent to the Lund Kennedy grading were done.^11^, ^12^ Age, gender, Lund MacKay score, symptoms, type of surgery were correlated with frequency of minor, major and serious ophthalmic complications. The surgery was performed by two senior surgeons. Surgical experience was rated from beginners (0–5 years) toward experienced more than 5 years. The ophthalmic complications are shown in Table 1.

**Table 1:** Rate of ophthalmic complications in total FESS.

Type of complication	No	%
*Minor*		
Injury to the lamina papyracea and periorbital ecchymosis and emphysema	5	0.3

*Major*		
Lacrimal duct injury	1	0.06

*Serious*	5	0.3
Retroorbital hematoma	2	0.12
Injury of optic nerve	2	0.12
Injury of orbital muscle	1	0.06

Surgery was performed under general anesthesia in all cases. A surgery consisted of polyp's removal with microdebrider, followed by middle metal antrostomy, partial or complete ethmoidectomy, frontal recess surgery and sphenoid surgery if necessary. After the surgery the middle meatus was packed with removable gauze packing for 7 days.

Statistical analysis was performed with Stat Soft Inc. (2011) Statistica software version no. 10. The Mann–Whitney *U* test and *x*^2^-test were used to evaluate differences between the positive or negative possibilities of complication. Univariate and multivariate analysis were done using logistic regression to obtain risk factors for ophthalmic complications of FESS. The level of significance was defined as *p* < 0.05.

## Results

The age ranged from 17 to 69 years (mean 45.6 years). Pathological findings and symptoms are shown in Table 2. The types of surgery are presented in Table 3. In our material 32.68% of the patients required revision surgery and only 10.1% had been done previously in our Department. The surgery procedures were done by two surgeons with dependent level of experience Table 4. All the patients with ophthalmic complications were diagnosed, treated and followed up by one senior clinical ophthalmologist. Overall complications occurred in 11 patients (0.66%). A minor complications was observed in 5 patients (0.3%) with the most frequent being periorbital ecchymosis with or without emphysema. Major ones were observed in one patient (0.06%) and were referred to lacrimal duct injury which was repaired during the same surgery. A serious complication occurred in 5 cases (0.3%) and referred to retroorbital hematoma (2 cases), optic nerve injury (2 cases) and one case of extraocular muscle injury. Among the group of serious complications in 2 cases of rtetroorbital hematoma we performed immediate orbital decompression with good results and complete recovery. In one case of extraocular muscle injury (medial rectus muscle) after the ophthalmic surgery correction and rehabilitation we do not achieved complete recovery and the patient has still diplopia. In both cases of optic nerve injury the decompression was done, but only in one case the visual acuity improved to 0.1 at Snellen chart. The second patient visual acuity was only hand motion. These 2 cases were qualified as permanent ophthalmic complications (0.12% of all surgeries).

**Table 2:** Pathophysiological findings and complications rate.

	*n*	Complications positive (*n* = 11)	Complications negative (*n* = 1647)	*p*-Value
Age (years)	45.6 ± 13.8	45.1 ± 12.3	47 ± 10.8	ns
Sex (male/female)	987/671	6/5	981/666	ns
Lund–MacKay score	12.4 ± 7.3	14.7 ± 8.9	9.4 ± 5.4	<0.028
Polyp score	2.6 ± 1.4	3.1 ± 1.5	1.7 ± 1.1	<0.05
Prevoius sinus surgery	542	9 ± 5.3	2 ± 1.1	<0.05
Treatment with anticoagulants	331	7 ± 3.8	4 ± 2.9	<0.05
CRS	675	3 ± 1.1	672 ± 15.6	ns
CRS with polyps	973	8 ± 3.4	965 ± 16.7	<0.05
Mucocele	10	0	10 ± 2.8	ns

ns, not significant.

**Table 3:** Type and extension of surgery in analyzed group of patients.

Surgery	*n*	%	Complications (*N*)	*p*-Value
Infundibulotomy	5	0.3	1	ns
Partial anterior ethmoidectomy	118	7.12	0	–
Complete ethmoidectomy	187	11.29	6	<0.05
Sphenoethmoidectomy	68	4.1	2	ns
Anterior frontoethmoidectomy	296	17.85	0	–
Complete frontoethmoidectomy	886	53.44	2	ns
Frontosphenoethmoidectomy	98	5.9	0	–
Total	1658	100	11	ns

ns, not significant.

**Table 4:** Percentage of operations done by beginners (0–5 years) and experienced (>5 years) surgeons with ophthalmic complications occurrence.

	0–5 years		>5 years	
	Surgeon 1	Surgeon 2	Surgeon 1	Surgeon 2
Number of cases	402	280	378	598
Complication (*N*) and type	3	2	3	3
Minor	0	1	2	2
Major	0	0	0	1
Serious	3	1	1	0
*p*-Value	ns	ns	ns	ns

ns, not significant.

## Discussion

Nevertheless, the incidence of ocular complications during ESS is rather low; they could be serious, leading to permanent dysfunction. The overall incidence of ESS complications are reported in several metaanalysis pointed its occurrence between 4.2–23% or 0.9–3.1%.^1^, ^3^, ^5^ There are only few analysis of orbital complications during ESS.^13^, ^14^ The incidence of this type of complications is range from 0.5% to 5%.^13^ In our investigation we tried to evaluate the frequency and types of ophthalmic complications using the novel system of classification to minor, major and serious proposed by Siedek.^1^ The exact incidence for orbital injury during ESS is unclear, but is still less than 1%.^1^, ^3^, ^5^, ^13^ The same result was obtained in our study, and the rate of all ophthalmology complications was 0.66%. The orbit and its content is at risk during ESS because the lamina papyracea is very thin or may be incomplete.^5^, ^6^, ^15^, ^16^ This site is the most potential risk area, especially when we do not have a good quality of vision or using powered instrumentation.^1^, ^3^, ^5^, ^6^, ^17^ The minor complications are referred to lamina papyracea injury mostly during maxillary antrostomy or ethmoidectomy. This complications are mostly seen with hypoplastic maxillary sinus or Silent Sinus Syndrome (SSS).^4^, ^5^, ^11^, ^13^, ^18^, ^19^ In this anatomic variants the uncinate very tightly connects to the lamina papyracea and should be resected with great attention.^5^, ^11^, ^13^ In our material we had one SSS and two cases of hypoplastic maxillary sinus which had been complicated with injury to the lamina papyracea leading to periorbital ecchymosis and in one case emphysema. The other two cases of minor complications occurred during ethmoidectomy after braking the lamina papyracea. All 5 cases (0.3%) do not need any intervention except standard postoperative treatment and control. The major complications as lacrimal duct injury is also connected with uncinectomy, if it is performed too far anterioly.^5^, ^13^ If the nasolacrimal duct will still drain, it is the best to leave it.^2^, ^5^, ^13^ In our one case (0.06%) during inspection of injured lacrimal duct we were not absolutely sure if it is open, so we performed dacryocystorhinostomy with silicon tube intubation. The most devastating orbital complications as orbital hematoma, optic nerve injury or external ocular muscle rupture could occurred during ethmoidectomy, sphenoethmoidectomy or frontoethmoidectomy.^2^, ^5^, ^13^ Orbital hematoma could developed as arterial injury (anterior or posterior ethmoid artery) or venous hemorrhage results from entry of the orbit through the lamina papyracea^1^, ^3^, ^5^, ^9^, ^13^ The incidence of this most common serious complication is about 0.12%, as was also observed in our study.^5^, ^9^ The hemorrhage can result in visual loss from optic nerve or retinal ischemia. This situation demanded very fast identification and urgent treatment. If the risk is low (low ocular pressure and vision is not compromised) medical treatment is adequate. In high ocular pressure and visual dysfunction immediate surgical intervention included lateral canthotomy, cantholysis and orbital decompression is recommended.^2^, ^3^, ^5^, ^13^ In most cases, as in our 2 patients (0.12%) after ophthalmology treatment and surgery the results are very good, with complete recovery.

Direct optic nerve injury is very rare and was found in our material in 2 cases (0.12%) as compared with literature review.^1^, ^3^, ^5^, ^13^ The nerve is commonly dehiscent in the sphenoid sinus or posterior ethmoid. The injury may be indirect (vascular) or direct (mechanical).^1^, ^3^, ^5^, ^13^, ^20^ In our work the injury was caused by hematoma and compression of the nerve in posterior ethmoid. In spite of intense intravenous corticosteroids and optic nerve decompression the results is not so satisfied.^1^, ^3^, ^5^ We obtained visual improvement in one case and no positive outcome in the second one. Also the injury of orbital muscle is one of the most devastating complication, frequently leading to permanent dysfunction.^1^, ^5^, ^6^, ^13^ Direct muscle transection is mostly seen with powered instrumentation surgery. The device extracts tissue very rapidly with very low tactile feedback to the surgeon about removable material.^1^, ^2^, ^14^ In our study we observed one patient (0.06%) with direct rectus muscle injury after microdebrider usage. Despite of ophthalmic surgery and rehabilitation the patient has still diplopia.

The risk factors for ESS complication depends on the extent of the disease and surgery, Lund–MacKay score, powered surgery, coexisting co morbidities, primary or revision surgery or surgeons experience.^3^, ^5^, ^6^, ^13^, ^21^ Asaka et al. reported that the risk depends on polyp score and asthma whereas the Lund–MacKay score did not.^3^ In our study univariate analysis found that polyp score, Lund–MacKay score, previous surgery and also anticoagulant treatment correlated significantly with the ophthalmic complications. We have to pointed that patients treated with anticoagulants had definitely worse quality of operative field due to more intense bleeding. Moreover, this patients more bleeds due to the blood pressure could not be lowered enough during the surgery.^1^ Multivariate analysis showed that only polyp score and pervious surgery correlated significantly with the occurrence of complications, whereas the Lund–MacKay score and anticoagulants did not (Table 5). As the extent of mucosal lesion influenced to clear vision for surgical landmarks the scaring in revision surgery much more changed it.^3^, ^21^ The surgeons experience did not influenced on complications rate, as it was also showed in other studies.^1^, ^3^, ^5^, ^6^ We can expect that surgeons who are more experienced are able to perform more difficult cases and fell more save with anatomical variants or powered devices. It is not truth, the same level of complications are observed at the beginners and experienced ones. Moreover, the rate of complications is not regard to the number of surgeries performed by single surgeon. On the other hand, when we operated the extent disease, especially nasal polyps we should performed not only total ethmoidectomies but also sphenoidectomy and frontoethomidectomy which are more predictable for unexpected complications. The management of these patients with pathology in close to the orbit could be associated with serious injuries, leading to permanent dysfunction. It was proved that the extent of the surgery also influenced to the rate of complications. There were observed especially in complete ethmoidectomies (*p* < 0.05).

**Table 5:** Risk factors for ophthalmic complications during endoscopic sinus surgery.

Univariate analysis	crud OR	95% CI	*p*-Value
Lund–MacKay score	1057	1.012–1.118	<0.021
Polyp score	1521	1.161–1.910	<0.016
Previous surgery	2031	1.576–2.114	<0.024
Anticoagulants	1594	1.478–1.810	<0.022

Multivariate analysis	Adjusted OR	95% CI	*p*-Value
Lund–MacKay score	1015	1.001–1.062	<0.562
Polyp score	1201	1.044–1.583	<0.030
Previous surgery	1902	1.246–1.671	<0.041
Anticoagulants	967	0.901–1.057	<0.638

## Conclusions

Orbital complications of endoscopic nasal surgery are rare, but could be potentially harmful. The incidence of serious complications, causing permanent disabilities is less than 0.3% but we should work to minimalize it. The most important parameters responsible for complications are extension of the disease, previous endoscopic surgery and coexisting anticoagulant treatment. Nevertheless, a keep going on CT reading, new devices and training are the best methods for save surgery.

## Conflicts of interest

The authors declare no conflicts of interest.
